# Supplementary material for: Genetic variant rs1205 is associated with COVID-19 outcomes: The Strong Heart Study and Strong Heart Family Study
Source: PLoS One. 2024 Apr 25;19(4):e0302464. doi: 10.1371/journal.pone.0302464 (PMC11045144; doi:10.1371/journal.pone.0302464)
Supplement: S1 Table — (DOCX) [file pone.0302464.s001.docx]

**Supplemental Table 1. Additional univariant associations with either fatal or non-fatal COVID-19.***

| Cohort | SNP | Risk allele | Model | Chi-square  p value** | OR*** | 95% CI | P value |
| --- | --- | --- | --- | --- | --- | --- | --- |
| SHFS | rs3091244 | G | Add | 0.325 | 1.415 | 0.881 - 2.272 | 0.151 |
| SHFS | rs3091244 | G | G-DOM | 0.198 | 2.472 | 0.594 - 10.279 | 0.213 |
| SHFS | rs3091244 | A | A-DOM | 0.257 | 0.717 | 0.402 - 1.278 | 0.259 |
| SHS | rs12329760 | C | Add | 0.132 | 0.668 | 0.435 - 1.024 | 0.064 |
| SHS | rs12329760 | C | C-DOM | 0.082 | 0.442 | 0.172 - 1.137 | 0.090 |
| SHS | rs12329760 | T | T-DOM | 0.136 | 1.519 | 0.873 - 2.645 | 0.139 |
| SHS | rs1800795 | G | Add | 0.238 | 0.729 | 0.394 - 1.350 | 0.315 |
| SHS | rs1800795 | G | G-DOM | 0.467 | NA | NA | NA |
| SHS | rs1800795 | C | C-DOM | 0.189 | 1.567 | 0.797 - 3.081 | 0.193 |
| SHS | rs8176719 | Ins | Add | 0.929 | 1.055 | 0.679 - 1.641 | 0.811 |
| SHS | rs8176719 | Ins | I-DOM | 0.915 | 1.031 | 0.591 - 1.799 | 0.915 |
| SHS | rs8176719 | Del | D-DOM | 0.700 | 0.816 | 0.290 - 2.297 | 0.701 |
| SHS | rs8176746 | G | Add | 0.628 | 1.958 | 0.487 - 7.876 | 0.344 |
| SHS | rs8176746 | G | G-DOM | 0.877 | NA | NA | NA |
| SHS | rs8176746 | T | T-DOM | 0.942 | 0.949 | 0.227 - 3.962 | 0.942 |
| SHS | rs1799752 | Ins | Add | 0.947 | 1.059 | 0.677 - 1.658 | 0.801 |
| SHS | rs1799752 | Ins | I-DOM | 0.746 | 1.214 | 0.374 - 3.942 | 0.746 |
| SHS | rs1799752 | Del | D-DOM | 0.881 | 0.958 | 0.551 - 1.667 | 0.881 |
| SHS | Blood type O |  | O vs Other | 0.899 | 0.965 | 0.553 - 1.683 | 0.899 |
| SHS | rs10735079 | A | Add | 0.126 | 1.342 | 0.794 - 2.270 | 0.272 |
| SHS | rs10735079 | A | A-DOM | 0.129 | 1.571 | 0.873 - 2.827 | 0.132 |
| SHS | rs10735079 | G | G-DOM | 0.336 | NA | NA | NA |
| SHS | rs1405655 | G | Add | 0.889 | 0.909 | 0.612 - 1.349 | 0.635 |
| SHS | rs1405655 | G | G-DOM | 0.732 | 0.903 | 0.502 - 1.623 | 0.732 |
| SHS | rs1405655 | A | A-DOM | 0.661 | 1.176 | 0.569 - 2.461 | 0.662 |
| SHS | rs1886814 | A | Add | 0.493 | 1.088 | 0.747 - 1.584 | 0.660 |
| SHS | rs1886814 | A | A-DOM | 0.350 | 1.327 | 0.732 - 2.407 | 0.351 |
| SHS | rs1886814 | G | G-DOM | 0.739 | 1.132 | 0.547 - 2.339 | 0.739 |
| SHS | rs2109069 | G | Add | 0.058 | 1.281 | 0.822 - 1.997 | 0.274 |
| SHS | rs2109069 | G | G-DOM | 0.773 | 1.086 | 0.620 - 1.903 | 0.773 |
| SHS | rs2109069 | A | A-DOM | 0.019 | 0.365 | 0.152 - 875 | 0.024 |
| SHS | rs9380142 | A | Add | 0.196 | 1.091 | 0.728 - 1.635 | 0.674 |
| SHS | rs9380142 | A | A-DOM | 0.697 | 0.897 | 0.517 - 1.554 | 0.697 |
| SHS | rs9380142 | G | G-DOM | 0.125 | 0.568 | 0.273 - 1.180 | 0.130 |
| SHS | rs111837807 | A | Add | 0.168 | 1.499 | 0.966 - 2.325 | 0.071 |
| SHS | rs111837807 | A | A-DOM | 0.060 | 1.689 | 0.973 - 2.930 | 0.062 |
| SHS | rs111837807 | G | G-DOM | 0.518 | 0.678 | 0.207 - 2.218 | 0.520 |
| SHS | rs2071351 | A | Add | 0.687 | 1.139 | 0.571 - 2.272 | 0.713 |
| SHS | rs2071351 | A | A-DOM | 0.602 | 1.213 | 0.586 - 2.513 | 0.603 |
| SHS | rs2071351 | G | G-DOM | 0.562 | NA | NA | NA |
| SHS | rs529565 | A | Add | 0.891 | 1.085 | 0.698 - 1.687 | 0.718 |
| SHS | rs529565 | A | A-DOM | 0.829 | 1.063 | 0.609 - 1.856 | 0.829 |
| SHS | rs529565 | G | G-DOM | 0.635 | 0.779 | 0.277 - 2.192 | 0.635 |
| SHS | rs10774671 | A | Add | 0.241 | 1.229 | 0.717 - 2.108 | 0.453 |
| SHS | rs10774671 | A | A-DOM | 0.261 | 1.406 | 0.774 - 2.555 | 0.263 |
| SHS | rs10774671 | G | G-DOM | 0.337 | NA | NA | NA |
| SHS | rs61667602 | A | Add | 0.677 | 0.656 | 0.245 - 1.756 | 0.402 |
| SHS | rs61667602 | A | A-DOM | 0.448 | 0.673 | 0.241 - 1.883 | 0.450 |
| SHS | rs61667602 | G | G-DOM | 0.535 | NA | NA | NA |

* Note: rs16944971 AF = 0.006 and rs7412 AF=0.02, so analysis was not performed.

** Pearson chi-square

*** OR: univariate logistic regression odds ratio
