# Supplementary material for: Genetic variant rs1205 is associated with COVID-19 outcomes: The Strong Heart Study and Strong Heart Family Study
Source: PLoS One. 2024 Apr 25;19(4):e0302464. doi: 10.1371/journal.pone.0302464 (PMC11045144; doi:10.1371/journal.pone.0302464)
Supplement: S2 Table — (DOCX) [file pone.0302464.s002.docx]

**Supplemental Table 2. Baseline hsCRP measures* in relation to rs1205 T-Dom genotype and COVID-19 case/control status.**

|  |  |  |  |  |  |  |  |
| --- | --- | --- | --- | --- | --- | --- | --- |
|  | **rs1205 T-Dom genotype** | | | **Alternative rs1205 genotype** | | |  |
| **hsCRP, mg/L** | **N** | **mean** | **SD** | **N** | **mean** | **SD** | **P value**** |
| **All values** | **2492** | **6.78** | **12.09** | **775** | **7.35** | **12.14** | **0.256** |
| **Excluding >10** | **2088** | **3.44** | **2.46** | **619** | **3.54** | **2.45** | **0.376** |
| **Excluding >15** | **2273** | **4.17** | **3.43** | **691** | **4.43** | **3.51** | **0.087** |
|  |  |  |  |  |  |  |  |
|  | **COVID-19 Case** | | | **COVID-19 Control** | | |  |
|  | **N** | **mean** | **SD** | **N** | **mean** | **SD** | **P value**** |
| **All values** | **85** | **5.78** | **5.46** | **3182** | **6.95** | **12.23** | **0.379** |
| **Excluding >10** | **72** | **3.83** | **2.36** | **2635** | **3.46** | **2.46** | **0.197** |
| **Excluding >15** | **80** | **4.76** | **3.60** | **2884** | **4.22** | **3.60** | **0.167** |

* Measured at SHS/SHFS phase before and closest to 2/1/20.

** Independent samples T-test
